# Deriving and understanding the risk of post-transplant recurrence of nephrotic syndrome in the light of current molecular and genetic advances

**DOI:** 10.1007/s00467-017-3793-2

**Published:** 2017-10-11

**Authors:** Agnieszka Bierzynska, Moin A. Saleem

**Affiliations:** Bristol Renal, and Children’s Renal Unit, University of Bristol, Bristol, BS1 3NY UK

**Keywords:** Podocyte, Nephrotic, Recurrence, Genetic, Transplantation

## Abstract

After renal transplantation, recurrence of the original disease is the second most common cause of graft loss, after rejection. The most dramatic manifestation of this phenomenon is in patients with nephrotic syndrome (NS). NS is a descriptive term describing a clinical picture centred on proteinuria arising from damage to the glomerular filtration barrier (GFB). There are many different drivers of that damage, ranging from immune dysregulation to genetic disorders and chronic disease/infections. The main categories in childhood are “idiopathic” (presumed immune mediated) and genetic NS, with further stratification of the idiopathic group according to steroid responses. A significant proportion of patients with NS progress to established renal failure, requiring transplantation, and one of the most difficult clinical scenarios faced by nephrologists is the recurrence of the original disease in up to 50% of patients, usually rapidly post-transplant. This is thought to be the archetypal “circulating factor” disease, in which as yet unknown circulating plasma “factor(s)” in the recipient target the donor kidney. The ability to predict in advance which patients will suffer recurrence would enhance our ability to counsel patients and families, and potentially identify those patients before transplant for tailored immunosuppressive preparation. Until very recently, stratification based on clinical categorisations has been poor in being able to predict those patients in whom disease will recur, and laboratory biomarkers are yet to be adequately refined. However, by mapping our growing understanding of disease mechanisms to clinical phenotypes, and with greatly improved genetic diagnostics, we have made progress in being able to stratify patients more specifically, and allow better predictive algorithms to be developed. Using our knowledge of podocyte biology, circulating factor-induced specific biomarkers are also being tested. This review is aimed at outlining those advances, and suggesting how we can move further forward in both clinical and biological markers of disease type.

## Introduction

Idiopathic nephrotic syndrome (INS) is one of the most common glomerular diseases in children and adults, the central event being podocyte injury. INS is a heterogeneous disease and treatment is largely empirical and unsuccessful, with steroids as the initial mainstay of therapy. Up to 90% of children with INS have some response to steroids and are labelled as steroid-“sensitive” (SSNS), and the rest as steroid-“resistant” (SRNS, most can also be described by histology as focal segmental glomerulosclerosis, FSGS), with single gene mutations underlying a large proportion of the latter group [1, 2]. The burden of morbidity is enormous, both to patients with lifelong chronic disease, and to health services, particularly managing dialysis and transplantation.

The current protocol for the management of INS is treatment with high-dose steroids. Of resistant patients, only 30% will respond over time to powerful second- and third-line immunosuppression; the rest suffer major long-term morbidity and renal failure requiring dialysis/transplantation. Up to 50% develop rapid recurrence post-transplantation, with eventual graft loss, despite highly intensive treatments.

Advances in genetics, both by identification of single gene mutations and in our ability to rapidly screen patients, have begun to allow practical steps towards the mechanistic stratification of disease and therefore of predicting disease recurrence in those patients. A patient identified as having a monogenic cause of their NS is far less likely to suffer recurrence of disease post-transplantation, although the absolute risk remains undefined. The challenge now is to enhance our understanding of which patients suffer recurrence, using our expanding knowledge of disease mechanisms based on podocyte biology.

## Predictions based on pre-transplant clinical categorisation

To date, different studies have identified different clinical features with relatively weak correlations with post-transplant recurrence. Features such as age at onset, race, live related donation, histological severity etc. have been weakly linked in some studies with an enhanced risk [3–6]. Odorico et al. retrospectively evaluated the effects of bilateral native nephrectomy before transplant in patients with recurrent disease post-transplant [7]. The incidence of recurrence was 40% in the nephrectomised patients as opposed to 16.1% among non-nephrectomised patients, although other small studies have not found a significant difference [8]. As a potential explanation, it was proposed that native kidneys act to absorb permeability factors, although I speculate that it is a reflection of the more aggressive circulating factor disease (CFD, see below).

The most consistent features reported in the literature over many years have been rapid progression to established renal failure (ERF), a lower age at diagnosis, and greater degrees of proteinuria in the recurrent groups [3, 6, 8, 9]. A summary of key findings regarding risk factors predisposing to recurrence, from the main studies in the literature, is provided in Table 1.

**Table 1:** Summary and comparison of clinical features in patients with and without recurrence of focal segmental glomerulosclerosis (FSGS) post-transplantation

Age group	Number of patients	Number with recurrence vs number with no recurrence	Age at diagnosis	Age at transplantation	Race: percentage white	Percentage male	Serum albumin at diagnosis (g/dL)	Genetic diagnosis	Urine protein at diagnosis	Pre-transplant proteinuria	Time to ERF	eGFR at diagnosis	Mesangial proliferation (native Bx)	Bilateral nephrectomy	Living donation	Reference
Paediatric	29	15 vs 14	NS	NS		NS					3.9 vs 6.2 years (*p* < 0.05)					[9]
Adult	22	5 vs 17	39 vs 48 years			20 vs 76 (*p* = 0.02)	2.63 vs 3.45 (*p* < 0.05)			7.0 vs 2.5 g/day (*p* < 0.05)	3.1 vs 11.9 years (*p* < 0.05)			3 vs 0 (*p* < 0.05)		[10]
Paediatric and adult	25	5 vs 20	12.5 vs 25.9 years (*p* = 0.02)								3.78 vs 5.68 years (NS)					[11]
Paediatric and adult	72	25 vs 53	NS			67 vs 73 (NS)					49 vs 101 months (*p* = 0.022)				52% vs 45% (NS)	[12]
Paediatric	16	6 vs 10	7.2 vs 6.1 years (NS)	11.8 vs 13.6 years (NS)		67 vs 90 (NS)					41 vs 77 months (*p* = 0.045)		29% vs 40% (NS)		71% vs 70% (NS)	[13]
Paediatric	132	27 vs 105			90 vs 59 (*p* = 0.027)	78 vs 48 (NS)										[5]
Paediatric and adult	59	13 vs 46	8.0 vs 9.1 years (children); 25.7 vs 28.4 years (adults)	50% recurrence in <15 years, 11% in adolescents/adults							3.5 vs 5.0 years (children, NS); 5.1 vs 4.8 years (adults, NS)					[14]
Adult	94	28 vs 66	23 vs 29 years	32 vs 38 (NS)		57 vs 53	20 vs 31 g/L (*p* < 0.01)		11.7 vs 5.9 g/day (*p* < 0.01)		4.9 vs 3.7 years (NS)			1 vs 5 (NS)	14% vs 22% (NS)	[6]
Adult	30	14 vs 16	31 vs 45 years (*p* < 0.03)			64 vs 81	13.4 vs 6.2 g/D (peak proteinuria, *p* < 0.01)	0% vs 70% (*p* < 0.01)			4.5 vs 5.5 years (NS)			69% vs 36% (*p* = 0.07)	24% vs 18% (NS)	[3]
Paediatric	22	9 vs 13	6.7 vs 5.3 years			56 vs 85 (NS)			9.0 vs 4.9 g/day (NS)		3.1 vs 6.1 years (*p* < 0.05)		44% vs 50% (NS)		44% vs 62% (NS)	[15]
Paediatric	150	57 vs 91			67 vs 82 (NS)	50 vs 72 (NS)		0% vs 27%			4.0 vs 3.0 years (NS)				15% vs 5% (*p* = 0.05)	[16]
Paediatric	28	6 vs 22	<6 vs >6 years (*p* < 0.05)		NS						NS		NS			[4]
Paediatric	13	8 vs 5	6.5 vs 2.9 years (NS)	6.9 vs 11.1 years (NS)		63 vs 100 (NS)	13.6 vs 1.9 g/D (peak proteinuria, NS)				13.2 vs 43.6 months (*p* < 0.05)					[8]

All data fields compare patients with recurrence with patients with no recurrence

*NS* non-significant, *ERF* established renal failure, *eGFR* estimated glomerular filtration rate

## Initial steroid sensitivity

We have recently reported by far the strongest predictive clinical feature of CFD to date, upon retrospective study of 150 grafts in FSGS children, from three large centres [16]. We hypothesised that the circulating factor is highly likely to be related to immune activation, and therefore if a patient responds to steroids early in the course of their disease (initial steroid sensitivity), this is proxy evidence for the presence of a circulating factor. Therefore, they are more likely to suffer from recurrence post-transplantation. Our study confirmed this hypothesis, showing that of 150 patients, 57 developed recurrence, and 26 out of 28 with initial steroid sensitivity suffered recurrence (*p* < 0.001, odds ratio 30). In contrast, none of the patients in the genetic or family history group suffered recurrence. This still leaves a clinically non-predictable group, those with primary steroid resistance and no genetic diagnosis according to the current screening. This group has an approximately 50% risk of recurrence, as shown by this study, and also according to our national screening study, which was far more complete in the genetic screening of the cohort [1].

## Secondary FSGS

Similar to genetic FSGS, FSGS secondary to other causes does not recur after kidney transplantation if the causes no longer exist; some of the reported FSGS cases without recurrence may in fact have been secondary FSGS. FSGS is an unspecific histological finding that is seen in many conditions of different aetiologies. Apart from genetic causes, which are considered to be primary, FSGS lesions can also be found as a secondary consequence of glomerular hypertrophy or hyperfiltration, toxins, obesity, HIV-associated nephropathy or scarring caused by previous injury (e.g., vasculitis, lupus).

Rudnicki reported that patients who present with proteinuria, but without oedema did not experience recurrence [17]. These patients would normally be categorised as having primary FSGS, as no underlying secondary cause was discovered. Therefore, the histological diagnosis of FSGS itself does not mean that the disease could recur after kidney transplantation.

The careful application of clinical criteria to separate disease categories is now beginning to clarify some of the risk features. In a study of 94 transplanted FSGS patients with a mean age of 37, Maas et al. separated patients into genetic (18 patients), secondary (10 patients) and idiopathic FSGS (66 patients). Only patients in the latter category developed recurrence, and the only independent predictor was serum albumin at diagnosis [6].

## Recurrence risk after re-transplantation

There is strong evidence that if a patient suffers a recurrence in the first allograft, then the second and subsequent transplants will have an even higher risk of recurrence compared with a first graft. The rate of recurrence is up to 80%, particularly if the first graft was lost early. Most studies have consistently quoted rates as high as 80% in the second transplant and >90% in the third and subsequent transplants [5, 18].

There is, however, some indication that if the recurrence in the first graft was relatively mild (i.e., the kidney was not lost rapidly), then subsequent grafts also follow the same pattern of recurrence with relatively prolonged function (ranging between 4 and 10.5 years in one study) [19].

## Synthesis of results from literature series

Overall, by reviewing all case series published, a certain pattern emerges. The first is from our own study, which shows by far the strongest predictor to date of recurrence, which is initial steroid sensitivity (alternatively termed secondary steroid resistance) [16]. This is further confirmed in our follow-up profile of a national cohort of SRNS patients, where 4 out of 5 secondary steroid-resistant patients (80%) developed recurrence, and 0 out of 25 patients with secondary resistance had a mutation in any of the 53 SRNS genes tested [1].

The second interesting trend is that patients with recurrence tend to have a lower serum albumin at presentation, greater proteinuria, and faster time to dialysis from presentation. This indicates more aggressive ongoing glomerular damage in (progressive) CFD, and could be used as an additional clinical clue early in the disease process. This would also explain the finding of a higher rate of bilateral nephrectomies in those who subsequently develop recurrence, as those with more aggressive disease are likely to be put forward for nephrectomy pre-transplant, to recover serum albumin levels.

Whole exome sequencing was performed on a UK national cohort of children with SRNS, and patients stratified according to the pattern of steroid response, followed by genetic diagnosis. Recurrence risk was highest in those with secondary steroid resistance, and lowest in those with a gene mutation underlying their SRNS.

With regard to the Kidney Disease Improving Global Outcomes (KDIGO) guidelines, the mean age at onset of NS and mean age at onset of end-stage renal failure (ESRF) were compared and the only significant difference (#) was noted for mean time to ESRF between primary + presumed monogenic and primary + presumed non-monogenic/unknown and secondary SRNS, with *p* value 0.0311 (two-tailed unpaired* t* test).

## Advances in biological understanding

The target cell of NS is the glomerular podocyte, and podocyte biology research has exploded in recent years. Landmark genetic and biological studies over the last 15 years have advanced glomerular biology at a remarkable pace, pointing compellingly to the podocyte as a uniquely functioning cell within the body, let alone the glomerulus, with pathways centring on the actin cytoskeleton and integrin signalling as tightly regulated nodes controlling the healthy function of the filtration barrier [20]. Idiopathic NS (INS) is an exemplar of primary glomerular disease. It is a rare disease, heterogeneous in cause, and therefore an accurate prediction of response post-transplant depends on stratification of disease at a mechanistic, rather than at an observational level.

1. Monogenic disease. Currently, there are single gene defects causing NS reported for 55 different genes [1, 21, 22]. Given the growing evidence that a monogenic cause for SRNS does not predispose to post-transplant recurrence, it is a crucial part of the management pathway to screen for known genetic mutations. Current availability of gene panels allows for this to be achieved at speed and relatively low cost compared with previous Sanger technologies (e.g., Bristol clinical SRNS gene panel, www.nbt.nhs.uk/severn-pathology/pathology-services/bristol-genetics-laboratory-bgl) [23].
2. “Circulating factor disease”. Recurrent disease post-transplant is the archetypal CFD, and is almost certainly linked to immune activation, and possibly a circulating factor(s) released by immune cells themselves. We currently have no reliable biomarkers to detect CFD, either pre- or post-transplant, although many have suggested potential circulating factors that would fit the biological scenario. These include suPAR [24], hemopexin, TNF-α [25], galactose [26], etc. To date, none has consistently been shown to be active in post-transplant disease.
3. Unknown. There is currently limited evidence either way to support whether there is a cohort of patients with INS with a different disease mechanism to 1 or 2 above. The discovery of biomarkers for CFD (see below), alongside complete genetic testing significantly clarify if such a unique phenotypic cohort exists, or what proportion of patients can be classified into the first two groups.

## Recurrence in NS caused by a monogenic disorder

To date, there has been little definitive evidence that patients with a genetic mutation causing SRNS will develop post-transplant recurrence of nephrotic range proteinuria. There are a few reports, predominantly with *NPHS2* variants, that suggest that this might occur. The risk of post-transplant recurrence in patients with podocin mutations may be rather confusing as single heterozygous mutations were included in some studies [27].

True homozygous or compound heterozygous mutations in podocin have been found in a few patients with post-transplant NS recurrence ranging from as soon as 7 days to 10 years post-transplant [27–34].

Similarly, post-transplant nephrotic-range proteinuria was noted in a patient with Frasier syndrome and a mutation in *WT1* [35]. FSGS recurrence has also been suggested in a patient with *ACTN4*, although the biopsy findings were non-specific [36].

As no anti-podocin antibodies have been detected in the tested *NPHS2* patients, even those with truncating mutations [27, 29, 30], the pathomechanism resulting in the disease recurrence is yet to be found. The pattern of proteinuria clearly needs to be carefully taken into account, as there are different potential causes in any transplant, and secondary morbidities should be considered.

## Re-analysis of SRNS causal variants

It is important that certain additional caveats are considered here, in particular, whether the variants reported as “causative” are truly so. A consistent issue in any gene test for mutation reporting is whether or not any rare variant is pathogenic, and there is no single definitive test. The proof lies predominantly in the frequency of that variant in the population (which also varies according to ethnicity), in addition to its frequency in the disease population, added to in silico corroborations such as conservation across species and deleterious functional predictions, and ultimately biological testing. Our ability to define variant frequencies in populations has expanded exponentially in recent years, in particular, with the development of large-scale reference datasets, such as the Exome Aggregation Consortium (ExAC). Interestingly, analysis of over 60,000 human exomes revealed that 72% of genes with predicted protein-truncating variants had no known human disease phenotype [37]. In addition, the average individual has 54 variants previously classified as causing a rare disorder, suggesting that these might have been incorrectly classified. When analysed, only 9 out of 192 variants previously classified as causing a Mendelian disease were supported as genuine by this new evidence of population variant frequency.

Overall, for SRNS, this suggests that almost all historical reports of causal variants need to be reassessed against current reference datasets, to filter out those that are no longer deemed causative. Equally, this means that historical reports of recurrence in “genetic” SRNS should be constantly reanalysed against this new information.

A particular misdiagnosis in the past is of pathogenicity of the R229Q variant in podocin, which is present in 4–5% of the general population, is associated with microalbuminuria [38], and is shown in biological models to have deleterious functional consequences [39]. More recently, however, it was elegantly shown that this variant only causes human SRNS when in combination with a pathogenic/rare variant on exon 8 of the second *NPHS2* allele [40]. Therefore, any recurrence seen in a patient with R229Q and a different *NPHS2* rare variant should be considered non-genetic unless an alternative pathogenic variant is identified.

A true genetic cause of recurrent CFD would likely involve a mutation in a gene related to the immune system, or a cognate receptor on the podocyte. It is interesting to note that we are not aware of any reports to date of familial recurrence (more than one member) of SRNS post-transplant, and advise caution in interpreting recurrence in cases with a structural podocyte defect.

## Which patients with SRNS should be offered living-related donation?

It has previously been shown in some studies that living-related donation (LD) is a risk factor for recurrence compared with cadaveric donation (CD). This is not consistent, and data from three large registries (NAPRTCS [41], USRDS [42] and RADR [43]) suggest no difference in recurrence rates between LD and CD recipients. However, the Australian and New Zealand Dialysis and Transplant Registry study did find that LD transplantation is an independent risk factor for recurrence (*p* = 0.02) [44]. We can speculate that in the studies showing a higher risk of LD, this is likely to represent a selection bias. The LD cohort historically will have screened out a proportion of genetic/familial donors, and is therefore enriched for recipients with CFD. Another factor may be the more rapid onset of renal failure in genetic vs non-genetic patients, meaning that genetic patients are more likely to be younger at transplantation, and therefore less suitable for an offer of an LD (adult) kidney.

To update advice on LD versus CD donation, we have divided the patients into those with an identified mutation, and those without. Individuals with identified autosomal dominant causality (heterozygous mutations) may present with variable phenotype/penetrance, including adult onset of NS; thus, living-related donors in this case may increase the risk of NS both in the recipient of the kidney and the donor, and should not be used.

In general, if a mutation is found in an autosomal recessive gene, a heterozygous carrier (parent) would be accepted to donate a kidney, with a negligible risk of recurrence. This excludes cases of Afro-Caribbean donors carrying the *APOL1* risk variant, where the risk of long-term renal decline is greater.

In cases in which a mutation has not been identified, our current practice is to strongly advise against living donation, unless the family is willing to proceed knowing the (high) risks. According to our recent national study [1], and supported by our retrospective review [16], the rate of recurrence in patients testing negative by a genetic screening panel [23] or exome screening is around 50%.

In cases in which there is a family history suggestive of a dominant mutation (with no responsible gene identified), it is clear that living donation from that side of the family should be avoided. Even if the potential donor does not have evident disease, the potential for incomplete penetrance remains.

## Circulating factor biomarkers

Circulating factor disease is a very significant subset of idiopathic nephrotic syndrome as a whole, and early post-transplant recurrence is an archetypal manifestation of CFD. Therefore, finding specific biomarkers in peripheral blood that identify CFD would be a major breakthrough in both early diagnosis and therefore targeted management, in addition to prediction of recurrence risk.

Savin et al. developed the original “permeability factor” assay, based on the swelling of ex vivo rat glomeruli in response to exposure to FSGS plasma [45]. Using this assay, a subsequent study reported 11 out of 13 children who tested positive for the permeability factor versus 4 out of 12 with negative results had a recurrence of FSGS after renal transplantation [9]. The odds ratio in the former group was 10.99 (with a 95% confidence interval of 1.6–75.5). However, using a different measure of glomerular permeability, another group failed to find any predictive value of pre-transplant measurements on the risk of recurrence [46].

Other groups have reported various putative circulating factors over many years, which have been reviewed elsewhere [47, 48], with no definitive candidate yet established.

Our approach over several years has been to utilise human podocytes in vitro and expose them to plasma exchange fluid taken from patients with early recurrence. This would mimic the disease situation of a circulating factor damaging podocytes in vivo, and the key is to find a damage pathway in the podocyte that is consistent in response to disease plasma. To date, we have demonstrated changes in the localisation of slit diaphragm proteins nephrin, podocin and CD2AP [49], in addition to enhanced phosphorylation of the actin regulating protein VASP [50], and functionally we can show an increase in podocyte motility. These responses are consistent when using plasma exchange fluid, although our follow-up testing of peripheral blood samples from patients at various stages of disease suggests that VASP phosphorylation may be less consistent as a biomarker (unpublished data). This could suggest different factors in pre-transplant disease, with differing downstream effects, although a more prosaic difficulty is obtaining fresh samples adequately stored. A key practical message in this type of clinical research is that aliquoting and early freezing of these valuable samples is as important a step in the experimental procedure as any other.

With these issues in mind, in the UK, we have undertaken a project termed the National Unified Renal Translational Research Enterprise (NURTuRE), in which samples will be collected according to strict protocols by dedicated research nursing staff, and stored centrally. This, and initiatives such as NEPTUNE in the USA [51], will be important resources in the future for high-quality biomarker studies in INS.

## What proportion of patients with SRNS have circulating factor disease?

We estimate, based on children with a recurrence rate in INS post-transplant of 40–60% (the archetypal CF disease), and a separate 20–30% rate of monogenic/familial disease in this cohort, there is a potential 10–40% “unknown mechanism” group remaining to be defined.

**Fig. 1:**
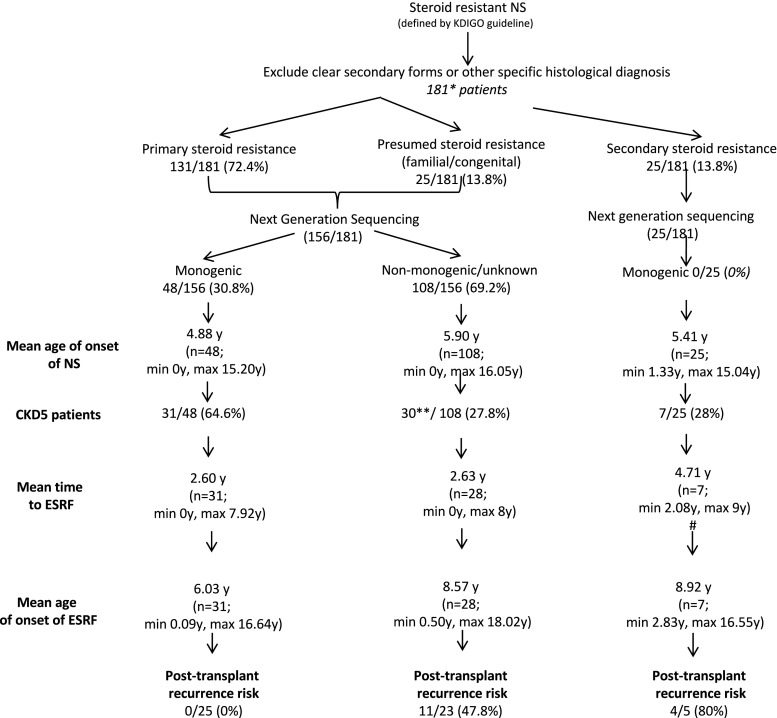
A genetic and clinical screening-based algorithm for predicting recurrence risk in steroid-resistant nephrotic syndrome (SRNS; from Bierzynska et al. [1], used with permission), *NS* nephrotic syndrome, *ESRF* end stage renal failure

Can we speculate what proportion of children overall with SRNS, and what proportion with primary SRNS in the “genetic testing negative” group have a CFD (and therefore are at risk of recurrence)? In our national cohort, tested by exome sequencing, the latter subset comprises 69% of all SRNS patients, and 48% of those transplanted from this subset suffered recurrence (Fig. 1) [1]. This implies that at least 48% of that subset has CFD, and potentially more, although those who did not suffer recurrence would have a different or milder disease phenotype. For the whole cohort, if we add that number to the secondary SRNS subgroup, that yields 79 out of 181 patients with presumed CFD, i.e. 43.6% of the total cohort. We know a separate 26.5% have a definite monogenic cause, leaving 29.9% overall unknown. Some of those have an undiscovered genetic mutation, and the rest could have CFD or another as yet unknown mechanism.

## Conclusion

Understanding and clinical identification of the mechanistic subgroups of NS lead to far better ability to predict outcomes, including recurrence risk. The group that suffer recurrence has a CFD that appears from the larger studies to date to be distinct from genetic NS. No familial cases of post-transplant recurrence have yet been reported.

With careful consideration of genetics, clinical features, and in future molecular biomarkers, we are getting far closer to accurate prognostication of the recurrence risk, and more targeted treatments to minimise that risk.

## References

[1] Bierzynska A, McCarthy HJ, Soderquest K, Sen ES, Colby E, Ding WY, Nabhan MM, Kerecuk L, Hegde S, Hughes D, Marks S, Feather S, Jones C, Webb NJ, Ognjanovic M, Christian M, Gilbert RD, Sinha MD, Lord GM, Simpson M, Koziell AB, Welsh GI, Saleem MA (2017). Genomic and clinical profiling of a national nephrotic syndrome cohort advocates a precision medicine approach to disease management. Kidney Int.

[2] Santin S, Bullich G, Tazon-Vega B, Garcia-Maset R, Gimenez I, Silva I, Ruiz P, Ballarin J, Torra R, Ars E (2011). Clinical utility of genetic testing in children and adults with steroid-resistant nephrotic syndrome. Clin J Am Soc Nephrol.

[3] Hickson LJ, Gera M, Amer H, Iqbal CW, Moore TB, Milliner DS, Cosio FG, Larson TS, Stegall MD, Ishitani MB, Gloor JM, Griffin MD (2009). Kidney transplantation for primary focal segmental glomerulosclerosis: outcomes and response to therapy for recurrence. Transplantation.

[4] Ingulli E, Tejani A (1991). Incidence, treatment, and outcome of recurrent focal segmental glomerulosclerosis posttransplantation in 42 allografts in children—a single-center experience. Transplantation.

[5] Tejani A, Stablein DH (1992). Recurrence of focal segmental glomerulosclerosis posttransplantation: a special report of the North American Pediatric Renal Transplant Cooperative Study. J Am Soc Nephrol.

[6] Maas RJ, Deegens JK, van den Brand JA, Cornelissen EA, Wetzels JF (2013). A retrospective study of focal segmental glomerulosclerosis: clinical criteria can identify patients at high risk for recurrent disease after first renal transplantation. BMC Nephrol.

[7] Odorico JS, Knechtle SJ, Rayhill SC, Pirsch JD, D'Alessandro AM, Belzer FO, Sollinger HW (1996). The influence of native nephrectomy on the incidence of recurrent disease following renal transplantation for primary glomerulonephritis. Transplantation.

[8] Fujisawa M, Iijima K, Ishimura T, Higuchi A, Isotani S, Yoshiya K, Arakawa S, Hamami G, Matsumoto O, Yoshikawa N, Kamidono S (2002). Long-term outcome of focal segmental glomerulosclerosis after Japanese pediatric renal transplantation. Pediatr Nephrol.

[9] Dall'Amico R, Ghiggeri G, Carraro M, Artero M, Ghio L, Zamorani E, Zennaro C, Basile G, Montini G, Rivabella L, Cardillo M, Scalamogna M, Ginevri F (1999). Prediction and treatment of recurrent focal segmental glomerulosclerosis after renal transplantation in children. Am J Kidney Dis.

[10] Sener A, Bella AJ, Nguan C, Luke PP, House AA (2009). Focal segmental glomerular sclerosis in renal transplant recipients: predicting early disease recurrence may prolong allograft function. Clin Transplant.

[11] PINTO J., LACERDA G., CAMERON J. S., TURNER D. R., BEWICK M., OGG C. S. (1981). RECURRENCE OF FOCAL SEGMENTAL GLOMERULOSCLEROSIS IN RENAL ALLOGRAFTS. Transplantation.

[12] Artero Mary, Biava Claude, Amend William, Tomlanovich Stephen, Vincenti Flavio (1992). Recurrent focal glomerulosclerosis: Natural history and response to therapy. The American Journal of Medicine.

[13] Cheong HI, Han HW, Park HW, Ha IS, Han KS, Lee HS, Kim SJ, Choi Y (2000). Early recurrent nephrotic syndrome after renal transplantation in children with focal segmental glomerulosclerosis. Nephrol Dial Transplant.

[14] Senggutuvan Prabha, Cameron J. Stewart, Hartley R. Barrie, Rigden Sue, Chantler Cyril, Haycock George, Williams D. Gwyn, Ogg Chisholm, Koffman Geoff (1990). Recurrence of focal segmental glomerulosclerosis in transplanted kidneys: Analysis of incidence and risk factors in 59 allografts. Pediatric Nephrology.

[15] Kim Sang Joon, Ha Jongwon, Jung In Mok, Ahn Moon Sang, Kim Minyoung, Lee Hyun Soon, Cheong Hae Il, Choi Yong (2001). Recurrent focal segmental glomerulosclerosis following renal transplantation in Korean pediatric patients. Pediatric Transplantation.

[16] Ding WY, Koziell A, McCarthy HJ, Bierzynska A, Bhagavatula MK, Dudley JA, Inward CD, Coward RJ, Tizard J, Reid C, Antignac C, Boyer O, Saleem MA (2014). Initial steroid sensitivity in children with steroid-resistant nephrotic syndrome predicts post-transplant recurrence. J Am Soc Nephrol.

[17] Rudnicki M (2016). FSGS recurrence in adults after renal transplantation. Biomed Res Int.

[18] Striegel JE, Sibley RK, Fryd DS, Mauer SM (1986). Recurrence of focal segmental sclerosis in children following renal transplantation. Kidney Int Suppl.

[19] Stephanian E, Matas AJ, Mauer SM, Chavers B, Nevins T, Kashtan C, Sutherland DE, Gores P, Najarian JS (1992). Recurrence of disease in patients retransplanted for focal segmental glomerulosclerosis. Transplantation.

[20] Welsh GI, Saleem MA (2012). The podocyte cytoskeleton—key to a functioning glomerulus in health and disease. Nat Rev Nephrol.

[21] Bierzynska A, Soderquest K, Dean P, Colby E, Rollason R, Jones C, Inward CD, McCarthy HJ, Simpson MA, Lord GM, Williams M, Welsh GI, Koziell AB, Saleem MA; NephroS; UK study of Nephrotic Syndrome (2016) MAGI2 mutations are responsible for congenital nephrotic syndrome. J Am Soc Nephrol 28(5):1614–1621 10.1681/ASN.2016040387 PMC5407720 27932480

[22] Prasad R, Hadjidemetriou I, Maharaj A, Meimaridou E, Buonocore F, Saleem M, Hurcombe J, Bierzynska A, Barbagelata E, Bergada I, Cassinelli H, Das U, Krone R, Hacihamdioglu B, Sari E, Yesilkaya E, Storr HL, Clemente M, Fernandez-Cancio M, Camats N, Ram N, Achermann JC, Van Veldhoven PP, Guasti L, Braslavsky D, Guran T, Metherell LA (2017). Sphingosine-1-phosphate lyase mutations cause primary adrenal insufficiency and steroid-resistant nephrotic syndrome. J Clin Invest.

[23] Sen Ethan S, Dean Philip, Yarram-Smith Laura, Bierzynska Agnieszka, Woodward Geoff, Buxton Chris, Dennis Gemma, Welsh Gavin I, Williams Maggie, Saleem Moin A (2017). Clinical genetic testing using a custom-designed steroid-resistant nephrotic syndrome gene panel: analysis and recommendations. Journal of Medical Genetics.

[24] Wei C, El Hindi S, Li J, Fornoni A, Goes N, Sageshima J, Maiguel D, Karumanchi SA, Yap HK, Saleem M, Zhang Q, Nikolic B, Chaudhuri A, Daftarian P, Salido E, Torres A, Salifu M, Sarwal MM, Schaefer F, Morath C, Schwenger V, Zeier M, Gupta V, Roth D, Rastaldi MP, Burke G, Ruiz P, Reiser J (2011). Circulating urokinase receptor as a cause of focal segmental glomerulosclerosis. Nat Med.

[25] Bitzan M, Babayeva S, Vasudevan A, Goodyer P, Torban E (2012). TNF alpha pathway blockade ameliorates toxic effects of FSGS plasma on podocyte cytoskeleton and beta 3 integrin activation. Pediatr Nephrol.

[26] Sgambat K, Banks M, Moudgil A (2013). Effect of galactose on glomerular permeability and proteinuria in steroid-resistant nephrotic syndrome. Pediatr Nephrol.

[27] Bertelli R, Ginevri F, Caridi G, Dagnino M, Sandrini S, Di Duca M, Emma F, Sanna-Cherchi S, Scolari F, Neri TM, Murer L, Massella L, Basile G, Rizzoni G, Perfumo F, Ghiggeri GM (2003). Recurrence of focal segmental glomerulosclerosis after renal transplantation in patients with mutations of podocin. Am J Kidney Dis.

[28] Billing H, Muller D, Ruf R, Lichtenberger A, Hildebrandt F, August C, Querfeld U, Haffner D (2004). NPHS2 mutation associated with recurrence of proteinuria after transplantation. Pediatr Nephrol.

[29] Becker-Cohen R, Bruschi M, Rinat C, Feinstein S, Zennaro C, Ghiggeri GM, Frishberg Y (2007). Recurrent nephrotic syndrome in homozygous truncating NPHS2 mutation is not due to anti-podocin antibodies. Am J Transplant.

[30] Weber S, Gribouval O, Esquivel EL, Moriniere V, Tete MJ, Legendre C, Niaudet P, Antignac C (2004). NPHS2 Mutation analysis shows genetic heterogeneity of steroid-resistant nephrotic syndrome and low post-transplant recurrence. Kidney Int.

[31] Hocker B, Knuppel T, Waldherr R, Schaefer F, Weber S, Tonshoff B (2006). Recurrence of proteinuria 10 years post-transplant in NPHS2-associated focal segmental glomerulosclerosis after conversion from cyclosporin a to sirolimus. Pediatr Nephrol.

[32] Ruf RG, Lichtenberger A, Karle SM, Haas JP, Anacleto FE, Schultheiss M, Zalewski I, Imm A, Ruf EM, Mucha B, Bagga A, Neuhaus T, Fuchshuber A, Bakkaloglu A, Hildebrandt F (2004). Patients with mutations in NPHS2 (podocin) do not respond to standard steroid treatment of nephrotic syndrome. J Am Soc Nephrol.

[33] Caridi G, Bertelli R, Perfumo F, Ghiggeri GM (2004). Heterozygous NPHS1 or NPHS2 mutations in responsive nephrotic syndrome and the multifactorial origin of proteinuria. Kidney Int.

[34] Caridi G, Bertelli R, Di Duca M, Dagnino M, Emma F, Onetti Muda A, Scolari F, Miglietti N, Mazzucco G, Murer L, Carrea A, Massella L, Rizzoni G, Perfumo F, Ghiggeri GM (2003). Broadening the spectrum of diseases related to podocin mutations. J Am Soc Nephrol.

[35] Ghiggeri GM, Aucella F, Caridi G, Bisceglia L, Ghio L, Gigante M, Perfumo F, Carraro M, Gesualdo L (2006). Posttransplant recurrence of proteinuria in a case of focal segmental glomerulosclerosis associated with WT1 mutation. Am J Transplant.

[36] Weins A, Kenlan P, Herbert S, Le TC, Villegas I, Kaplan BS, Appel GB, Pollak MR (2005). Mutational and biological analysis of alpha-actinin-4 in focal segmental glomerulosclerosis. J Am Soc Nephrol.

[37] Lek M, Karczewski KJ, Minikel EV, Samocha KE, Banks E, Fennell T, O’Donnell-Luria AH, Ware JS, Hill AJ, Cummings BB, Tukiainen T, Birnbaum DP, Kosmicki JA, Duncan LE, Estrada K, Zhao F, Zou J, Pierce-Hoffman E, Berghout J, Cooper DN, Deflaux N, DePristo M, Do R, Flannick J, Fromer M, Gauthier L, Goldstein J, Gupta N, Howrigan D, Kiezun A, Kurki MI, Moonshine AL, Natarajan P, Orozco L, Peloso GM, Poplin R, Rivas MA, Ruano-Rubio V, Rose SA, Ruderfer DM, Shakir K, Stenson PD, Stevens C, Thomas BP, Tiao G, Tusie-Luna MT, Weisburd B, Won HH, Yu D, Altshuler DM, Ardissino D, Boehnke M, Danesh J, Donnelly S, Elosua R, Florez JC, Gabriel SB, Getz G, Glatt SJ, Hultman CM, Kathiresan S, Laakso M, McCarroll S, McCarthy MI, McGovern D, McPherson R, Neale BM, Palotie A, Purcell SM, Saleheen D, Scharf JM, Sklar P, Sullivan PF, Tuomilehto J, Tsuang MT, Watkins HC, Wilson JG, Daly MJ, MacArthur DG, Exome Aggregation C (2016). Analysis of protein-coding genetic variation in 60,706 humans. Nature.

[38] Pereira AC, Pereira AB, Mota GF, Cunha RS, Herkenhoff FL, Pollak MR, Mill JG, Krieger JE (2004). NPHS2 R229Q Functional variant is associated with microalbuminuria in the general population. Kidney Int.

[39] Tsukaguchi H, Sudhakar A, Le TC, Nguyen T, Yao J, Schwimmer JA, Schachter AD, Poch E, Abreu PF, Appel GB, Pereira AB, Kalluri R, Pollak MR (2002). NPHS2 mutations in late-onset focal segmental glomerulosclerosis: R229Q is a common disease-associated allele. J Clin Invest.

[40] Tory K, Menyhard DK, Woerner S, Nevo F, Gribouval O, Kerti A, Straner P, Arrondel C, Huynh Cong E, Tulassay T, Mollet G, Perczel A, Antignac C (2014). Mutation-dependent recessive inheritance of NPHS2-associated steroid-resistant nephrotic syndrome. Nat Genet.

[41] Baum MA, Stablein DM, Panzarino VM, Tejani A, Harmon WE, Alexander SR (2001). Loss of living donor renal allograft survival advantage in children with focal segmental glomerulosclerosis. Kidney Int.

[42] Abbott KC, Sawyers ES, Oliver JD, Ko CW, Kirk AD, Welch PG, Peters TG, Agodoa LY (2001). Graft loss due to recurrent focal segmental glomerulosclerosis in renal transplant recipients in the United States. Am J Kidney Dis.

[43] Hariharan S, Adams MB, Brennan DC, Davis CL, First MR, Johnson CP, Ouseph R, Peddi VR, Pelz C, Roza AM, Vincenti F, George V (1999). Recurrent and de novo glomerular disease after renal transplantation: a report from renal allograft disease registry. Transplant Proc.

[44] Francis A, Trnka P, McTaggart SJ (2016). Long-term outcome of kidney transplantation in recipients with focal segmental glomerulosclerosis. Clin J Am Soc Nephrol.

[45] Savin VJ, Sharma R, Sharma M, McCarthy ET, Swan SK, Ellis E, Lovell H, Warady B, Gunwar S, Chonko AM, Artero M, Vincenti F (1996). Circulating factor associated with increased glomerular permeability to albumin in recurrent focal segmental glomerulosclerosis. N Engl J Med.

[46] Le Berre L, Godfrin Y, Lafond-Puyet L, Perretto S, Le Carrer D, Bouhours JF, Soulillou JP, Dantal J (2000). Effect of plasma fractions from patients with focal and segmental glomerulosclerosis on rat proteinuria. Kidney Int.

[47] Konigshausen E, Sellin L (2016). Circulating permeability factors in primary focal segmental glomerulosclerosis: a review of proposed candidates. Biomed Res Int.

[48] Kronbichler A, Saleem MA, Meijers B, Shin JI (2016). Soluble urokinase receptors in focal segmental glomerulosclerosis: a review on the scientific point of view. J Immunol Res.

[49] Coward RJ, Foster RR, Patton D, Ni L, Lennon R, Bates DO, Harper SJ, Mathieson PW, Saleem MA (2005). Nephrotic plasma alters slit diaphragm-dependent signaling and translocates nephrin, podocin, and CD2 associated protein in cultured human podocytes. J Am Soc Nephrol.

[50] Harris JJ, McCarthy HJ, Ni L, Wherlock M, Kang H, Wetzels JF, Welsh GI, Saleem MA (2013). Active proteases in nephrotic plasma lead to a podocin-dependent phosphorylation of VASP in podocytes via protease activated receptor-1. J Pathol.

[51] Gadegbeku CA, Gipson DS, Holzman LB, Ojo AO, Song PX, Barisoni L, Sampson MG, Kopp JB, Lemley KV, Nelson PJ, Lienczewski CC, Adler SG, Appel GB, Cattran DC, Choi MJ, Contreras G, Dell KM, Fervenza FC, Gibson KL, Greenbaum LA, Hernandez JD, Hewitt SM, Hingorani SR, Hladunewich M, Hogan MC, Hogan SL, Kaskel FJ, Lieske JC, Meyers KE, Nachman PH, Nast CC, Neu AM, Reich HN, Sedor JR, Sethna CB, Trachtman H, Tuttle KR, Zhdanova O, Zilleruelo GE, Kretzler M (2013). Design of the Nephrotic Syndrome Study Network (NEPTUNE) to evaluate primary glomerular nephropathy by a multidisciplinary approach. Kidney Int.

